# A bagging dynamic deep learning network for diagnosing COVID-19

**DOI:** 10.1038/s41598-021-95537-y

**Published:** 2021-08-11

**Authors:** Zhijun Zhang, Bozhao Chen, Jiansheng Sun, Yamei Luo

**Affiliations:** 1 School of Automation Science and Engineering, South China University of Technology, Guangzhou, 510640 China; 2 Guangdong Artificial Intelligence and Digital Economy Laboratory (Pazhou Lab), Guangzhou, 510335 China; 3 School of Automation Science and Engineering, East China Jiaotong University, Nanchang, 330052 China; 4 Shaanxi Provincial Key Laboratory of Industrial Automation, School of Mechanical Engineering, Shaanxi University of Technology, Hanzhong, 723001 China; 5 School of Information Technology and Management, Hunan University of Finance and Economics, Changsha, 410205 China

**Keywords:** Computational science, Diseases

## Abstract

COVID-19 is a serious ongoing worldwide pandemic. Using X-ray chest radiography images for automatically diagnosing COVID-19 is an effective and convenient means of providing diagnostic assistance to clinicians in practice. This paper proposes a bagging dynamic deep learning network (B-DDLN) for diagnosing COVID-19 by intelligently recognizing its symptoms in X-ray chest radiography images. After a series of preprocessing steps for images, we pre-train convolution blocks as a feature extractor. For the extracted features, a bagging dynamic learning network classifier is trained based on neural dynamic learning algorithm and bagging algorithm. B-DDLN connects the feature extractor and bagging classifier in series. Experimental results verify that the proposed B-DDLN achieves 98.8889% testing accuracy, which shows the best diagnosis performance among the existing state-of-the-art methods on the open image set. It also provides evidence for further detection and treatment.

## Introduction

COVID-19 has been rapidly and widely spreading as an ongoing pandemic throughout the world since the end of 2019 and has been responsible for a high number of fatalities^1–3^. According to statistics on the Worldometers.info website, over 198 million people have been diagnosed with COVID-19 and about 4.22 million deaths have occurred up to the end of July, 2021. Most detection methods for COVID-19, including nucleic acid amplification testing, rely on pathogen testing^4^. However, pathogen testing has some limitations. First of all, it requires testing kits that have limited availability in the supply chain^5^. Secondly, it is time-consuming because the detection steps are tedious and have high technical requirements^6,7^.

Given the limitations of nucleic acid amplification testing, the recognition of COVID-19 indications in X-ray chest radiography images can be performed to identify COVID-19 patients, which makes the diagnosis of the disease and its severity more intuitive^8,9^. This is a vital verification method with simple operation. The equipments required for X-ray chest radiography are lightweight and transportable^10^. The entire recognition process takes much less time (about 15 s per patient^10^) and manual operations to give the final visualized results, thus providing considerable assistance in a convenient manner to clinicians in practice^11^. Especially in China, many cases can be identified as suspected COVID-19 infections if the characteristic manifestations are observed in X-ray scans^11,12^. In the field of intelligent medical diagnosis, deep learning models have been widely developed and applied in recent years. They give their excellent abilities of feature extraction and classification^13^. An increasing number of researchers have applied deep learning and computer vision technologies to diagnose COVID-19 by recognizing medical images^14^. For example, Zhang et al. proposed a confidence-aware anomaly detection model that consists of a shared feature extractor, an anomaly detection module, and a confidence prediction module. The model achieved an AUC of 0.8361 and a sensitivity of 0.7170^15^. To further improve the diagnostic effect, Majeed et al. designed a convolutional neural network (CNN) model for COVID-19 detection from X-ray chest radiography images that obtained 0.9315 sensitivity and 0.9786 specificity^16^. Singh et al. proposed a deep convolutional neural network, whose hyper-parameters can be tuned by using multi-objective adaptive differential evolution. Their model achieved $94.48\%$ testing accuracy^17^. To accelerate diagnosis, Brunese et al. considered a deep learning network based on a VGG-16 model by exploiting transfer learning with an average time of approximately 2.5 s and $97.00\%$ average accuracy^18^. On the face of it, the accuracy and related performance indicate a great diagnosis level with a low misdiagnosis rate. However, without shortcut connections in these deep learning models, the vanishing-gradient problem may occur during the training process.

To effectively solve the vanishing-gradient problem, He et al. proposed a residual convolutional neural network (ResNet) in 2015 by adding shortcut connections and enhancing training efficiency efficiency^19–22^. Inspired by the method in reference^20^, Luz et al. applied ResNet50 to COVID-19 detection in X-ray images, which achieved $93.9\%$ overall accuracy, $96.8\%$ sensitivity, and $100\%$ positive prediction^23^. Based on the fine-tuned ResNet model, Farooq et al. developed a deep learning framework called COVID-ResNet to screen COVID-19 from radiographs that achieved $96.23\%$ accuracy and $100\%$ sensitivity with only 41 epochs^24^. For further developments of the ResNet, densely connected convolutional networks (DenseNet) was proposed in 2017 by designing dense blocks, which alleviated the vanishing-gradient problem, and strengthened feature propagation, thus encouraging feature reuse and substantially reducing the number of parameters effectively^25^. Based on DenseNet, Wang et al. designed and proposed COVID-Net architecture for COVID-19 diagnosis that achieved $93.33\%$ accuracy by recognizing X-ray images of normal, COVID-19, and ordinary pneumonia cases^26^. To improve the models’ initial performance, Narayan Das et al. combined deep transfer learning with the Xception model for COVID-19 diagnosis, which could achieve $97.41\%$ testing accuracy^27^. However, the generalization performance of fully connected layer at the end of ResNet-based models may not be strong enough to discriminate and classify deep convolutional features^28^.

For improving the generalization performance of the classifier, support vector machine (SVM) is an appropriate choice to classify samples precisely by searching for the optimal decision boundary. Researchers have combined CNN and SVM for image recognition, where CNN is applied for feature extraction and SVM classifies deep extracted features. For COVID-19 diagnosis, Sethy et al. used SVM to classify deep image features extracted from the fully connected layer of ResNet50, which achieved $95.38\%$ diagnosis accuracy^29^. To enhance the classification efficiency, Novitasari et al. removed the fully connected layers of the pre-trained ResNet18 as a feature extractor. For the image features extracted from the global average pooling layer, they utilized principal component analysis and relief methods in feature selection and applied SVM as a classifier, which achieved $96.15\%$ diagnosis accuracy^30^. However, for the nonlinear distribution of samples, we should find an appropriate kernel function to map these samples into linear separated space, which requires too many computational resources and too much model training time.

To accelerate training models with less computational overhead, neural dynamic learning algorithm (NDLA) has been proposed under the restrictions of high real-time requirement and limited hardware resources. With the characteristics of error exponential convergence and parallel computing, NDLA has been used to train some neural networks. For example, in order to solve time-varying convex quadratic programming problems constrained by linear equality and obtain online solutions to the time-varying Sylvester equation, a varying-parameter convergent-differential neural network (VP-CDNN) was proposed by using an exponential-type time varying design function^31,32^. Related theoretical analysis has proved that using VP-CDNN can make the residual error over the continuous time converge to zero super-exponentially. Based on the proposed VP-CDNN, various practical problems in time-varying over-determined system^33^, time-varying complex Sylvester equation^34^, disturbed time-varying inversion systems^35^, robot tracking^36,37^, and unmanned aerial vehicle controller^38^ have been effectively solved. Zhang et al. designed adaptive multi-layer neural dynamics-based controllers of multi-rotor unmanned aerial vehicles that can adapt to various complex transportation needs^39^. In addition, to solve the time-varying quadratic programming problem and robot tracking problem, a power-type varying-parameter recurrent neural network was proposed that is different from VP-CDNN^40,41^. To solve the non-repetitive motion problem of redundant robot manipulators, Zhang et al. proposed an adaptive fuzzy recurrent neural network that can avoid the saturation of time-varying design functions^42^. For application in machine learning and pattern recognition, a voting convergent difference neural network was proposed for diagnosing the presence of breast cancer and breast tumor types that achieved $100\%$ diagnosis accuracy^43^. Thus, NDLA has faster convergence rate and higher accuracy^33^, and a network trained by an NDLA is more suitable for solving problems that have challenging real-time requirements with limited hardware resources. However, to the best of the authors’ knowledge, NDLA-based networks have not yet been applied in the field of deep learning and image classification.

For a faster and more accurate COVID-19 diagnosis, this paper proposes a bagging dynamic deep learning network (B-DDLN) that consists of two modules: a feature extractor and a bagging classifier. In the proposed B-DDLN, five pre-trained convolution blocks are used as a feature extractor for X-ray chest radiography images and a bagging classifier based on NDLA and bagging algorithm is applied to classify these image features. The structure of the bagging classifier is lightweight and its cost function is more easily designed in comparison to most classifiers. The proposed B-DDLN can rapidly make the training and testing errors converge and enhance the diagnosis precision. Owing to the utilization of a bagging classifier, B-DDLN can solve class-imbalance problems by random under-sampling and further enhance the diagnosis results’ accuracy and reliability.

## Bagging dynamic deep learning network

In this section, the proposed bagging dynamic deep learning network (B-DDLN) is designed and analyzed detailedly in four stages. First of all, the construction of the B-DDLN is proposed. Secondly, the principle of dynamic learning network are presented for designing a classifier. Thirdly, neural dynamic learning algorithm (NDLA) of a dynamic learning network is derived in detail. Fourthly, based on bootstrap aggregation and the voting decision method among several dynamic learning networks trained by NDLA with various mapping functions, a bagging dynamic learning network classifier is proposed to enhance the generalization performance of a dynamic learning network.

### Construction of B-DDLN diagnosis model

For the application of medical image classification, a convolutional neural network is very effective because of its convolution blocks for extracting differentiated image features^19,20^. However, the generalization performance of fully connected layers in CNNs may not be strong enough to discriminate and classify deep convolutional features^28^. For diagnosing COVID-19, the accuracy and precision cannot be stabilized at a satisfactory level when only a CNN is used. To enhance the generalization performance of diagnosis model, B-DDLN is proposed in this paper, which consists of two modules: a feature extractor and a bagging classifier. In the feature extractor, the convolution blocks consist of convolutional layers, pooling layers, batch normalization, ReLU layers and shortcut connections. They are designed and pre-trained as a whole feature extractor for X-ray chest radiography images, and bagging dynamic learning network classifier based on NDLA and bagging algorithm is applied to classify these image features and generate diagnostic results.

**Figure 1:**
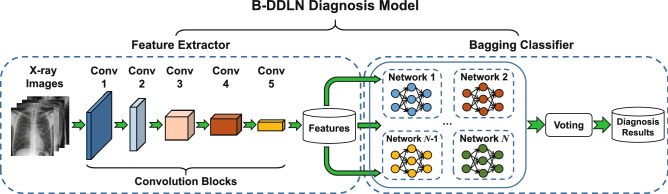
Construction of the proposed bagging dynamic deep learning network (B-DDLN) diagnosis model. The convolution blocks consist of convolutional layers, pooling layers, batch normalization, ReLU layers, and shortcut connections. In the feature extractor, five convolution blocks are designed and pre-trained for extracting the features of X-ray chest radiography images, and a bagging dynamic learning network classifier that consists of *N* unit dynamic learning networks is responsible for recognizing these features to generate the final diagnosis results.

Figure 1 shows how the proposed B-DDLN is constructed. We use images from training set to pre-train five designed convolution blocks as a feature extractor. Suppose a three-channel image tensor ${\varvec{g}}_{s}\in {\mathbb {R}}^{224\times 224\times 3}$ is input into this feature extractor, its corresponding feature vector $h({\varvec{g}}_{s})\in {\mathbb {R}}^{1\times 512}$ can be obtained. After gathering all the features of the training images from the feature extractor, we use these features to train the bagging dynamic learning network classifier and evaluate the complete proposed B-DDLN. The bagging dynamic learning network classifier consists of *N* dynamic learning networks, where *N* denotes the number of dynamic learning networks. These *N* dynamic learning networks are trained by using *N* subsets randomly collected from all the extracted training feature samples and NDLA with *N* different types of mapping functions.

As the proposed B-DDLN diagnosis model combines convolution blocks as a feature extractor and bagging dynamic learning network classifier, the constructed principle is termed B-DDLN constructed algorithm. Algorithm 1 illustrates the steps in detail. 
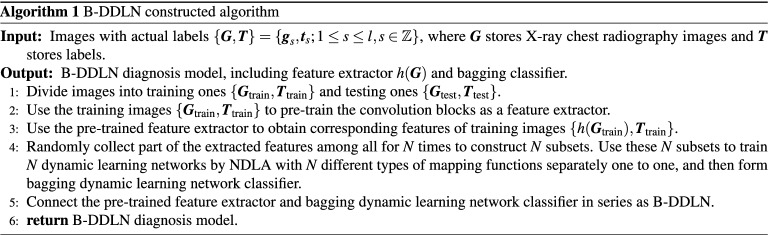


### Topology and principle of dynamic learning network

A three-layer dynamic learning network is designed and proposed in the bagging classifier module of the proposed B-DDLN. Some variables are explained as below:

- *l*: Number of input samples.
- *m*: Feature dimensions.
- *n*: Number of hidden neurons.
- *q*: Number of classes for samples.
- ${{\varvec{X}}}$: An $l\times m$ input matrix storing *l* samples with *m* dimensions. $x_{si}(1\le s\le l,1\le i\le m;s,i\in {\mathbb {Z}})\in {{\varvec{X}}}$ represents the value of the $i{\text {th}}$ dimension for the $s{\text {th}}$ sample.
- ${{\varvec{V}}}$: An $m\times n$ matrix storing weights connecting input and hidden layers. $v_{ij}(1\le i\le m,1\le j\le n;i,j\in {\mathbb {Z}})\in {{\varvec{V}}}$ represents the weight component connecting the $i{\text {th}}$ input neuron with the $j{\text {th}}$ hidden neuron.
- ${{\varvec{I}}}$: An $l\times n$ matrix input into the hidden layer.
- ${{\varvec{Q}}}$: An $l\times n$ hidden output matrix correspond to ${{\varvec{I}}}$.
- ${{\varvec{W}}}$: An $n\times q$ matrix storing weights connecting hidden and output layers. $w_{jr}(1\le j\le n,1\le r\le q;j,r\in {\mathbb {Z}})\in {{\varvec{W}}}$ represents the weight component connecting the $j{\text {th}}$ hidden neuron with the $r{\text {th}}$ output neuron.
- ${{\varvec{Y}}}$: An $l\times q$ predicted diagnosis output matrix.
- $f _{j}(\cdot )(1\le j\le n;j\in {\mathbb {Z}})$: Activation functions in the $j{\text {th}}$ hidden neurons.
- $g (\cdot )$: Activation function in the output neurons.
- ${\varvec{{\bar{Y}}}}$, ${{\varvec{L}}}$: $l\times q$ label matrices, where ${\varvec{\bar{Y}}}$ is encoded as $-1$-1 format and ${{\varvec{L}}}$ is encoded by one-hot vectors.
- ${{\varvec{P}}}$: An $l\times q$ class possibility matrix calculated by softmax formula.
- $\varepsilon$: Training error of dynamic learning network calculated by cross entropy formula.

The feed-forward output of the dynamic learning network is formulated as

1 $$\begin{aligned} {{\varvec{Y}}}=g ({{\varvec{Q}}}{{\varvec{W}}})=g ({{\varvec{F}}}({{\varvec{I}}}){{\varvec{W}}})\in {\mathbb {R}}^{l\times q} \end{aligned}$$

where

2 $$\begin{aligned} {{\varvec{X}}}&= {} \left[ \begin{array}{lll}{x_{11}} &{} {\cdots } &{} {x_{1m}} \\ {\vdots } &{} {\ddots } &{} {\vdots } \\ {x_{l1}} &{} {\cdots } &{} {x_{lm}}\end{array}\right] \in {\mathbb {R}}^{l\times m} \end{aligned}$$

3 $$\begin{aligned} {{\varvec{Q}}}&= {} {{\varvec{F}}}({{\varvec{I}}})={{\varvec{F}}}({{\varvec{X}}}{{\varvec{V}}})\in {\mathbb {R}}^{l\times n} \end{aligned}$$

4 $$\begin{aligned} {{\varvec{F}}}({{\varvec{I}}})&= {} \left[ f_{1}({{\varvec{i}}}_{1});f_{2}({{\varvec{i}}}_{2});f_{3}({{\varvec{i}}}_{3}); \ldots ;f_{j}({{\varvec{i}}}_{j});\ldots ;f_{n}({{\varvec{i}}}_{n})\right] \end{aligned}$$

5 $$\begin{aligned} {{\varvec{W}}}&= {} \left[ \begin{array}{lll}{w_{11}} &{} {\cdots } &{} {w_{1q}} \\ {\vdots } &{} {\ddots } &{} {\vdots } \\ {w_{n1}} &{} {\cdots } &{} {w_{nq}}\end{array}\right] \in {\mathbb {R}}^{n\times q}. \end{aligned}$$

In Eq. (4), ${{\varvec{i}}}_{j}\in {\mathbb {R}}^{l\times 1}$ denotes the $j{\text {th}}$ column vector of ${{\varvec{I}}}\in {\mathbb {R}}^{l\times n}$. For activation functions in the hidden and output neurons, two cases are listed as examples, i.e.,

- Case 1: All the activation functions are set as softsign function. The expression is
  6 $$\begin{aligned} f_{j}(z)=\textit{g}(z)=\frac{z}{1+|z|}(1\le j\le n;j\in {\mathbb {Z}}). \end{aligned}$$
- Case 2: Activation functions of the output neurons are still set as softsign function, while those of the hidden neurons are power-softsign function. The expression of power-softsign function is
  7 $$\begin{aligned} f_{j}(z)=\left( \frac{z}{1+|z|} \right) ^{j-1}(1\le j\le n;j\in {\mathbb {Z}}). \end{aligned}$$

For the $s{\text {th}}$ sample ${{\varvec{x}}}_{s}=\left[ {x_{s1}},\ldots , x_{sm}\right] \in {\mathbb {R}}^{1\times m}$ ($1\le s\le l;s\in {\mathbb {Z}}$) from ${{\varvec{X}}}$, the output through dynamic learning network ${{\varvec{y}}}_{s}=\left[ y_{s1}, \ldots , y_{sq}\right] \in {\mathbb {R}}^{1\times q}$ is calculated using Eq. (1), and the corresponding class probabilities vector ${{\varvec{P}}}_{s}$ is obtained using the softmax formula

8 $${\varvec {P}}_{s} = \left[ {P_{{s1}} , \ldots ,P_{{sq}} } \right] = \left[ {\begin{array}{*{20}l} {\frac{{\exp (y_{{s1}} )}}{{\sum\nolimits_{{r = 1}}^{q} {\exp (y_{{sr}} )} }}, \ldots ,\frac{{\exp (y_{{sq}} )}}{{\sum\nolimits_{{r = 1}}^{q} {\exp (y_{{sr}} )} }}} \hfill \\ \end{array} } \right] \in \mathbb{R}^{{1 \times q}}$$

where $P_{s1},\ldots ,P_{sq}\in [0,1],\sum \limits _{r=1}^{q}P_{sr}=1$. If $P_{sr}=\max \left\{ P_{s1},\ldots ,P_{sq}\right\} (1\le r\le q;r\in {\mathbb {Z}})$, ${{\varvec{x}}}_{s}$ is predicted as belonging to the $r{\text {th}}$ class according to Bayesian decision principle based on the minimum classification error probability^44^. Similarly, the class probability matrix ${{\varvec{P}}}$ for *l* input samples is calculated below.

9 $${{\varvec{P}}} = \left[ {\begin{array}{*{20}l} {P_{{11}} } \hfill & \cdots \hfill & {P_{{1q}} } \hfill \\ \vdots \hfill & \ddots \hfill & \vdots \hfill \\ {P_{{l1}} } \hfill & \cdots \hfill & {P_{{lq}} } \hfill \\ \end{array} } \right] = \left[ {\begin{array}{*{20}l} {\frac{{\exp (y_{{11}} )}}{{\sum\nolimits_{{r = 1}}^{q} {\exp (y_{{1r}} )} }}} \hfill & \cdots \hfill & {\frac{{\exp (y_{{1q}} )}}{{\sum\nolimits_{{r = 1}}^{q} {\exp (y_{{1r}} )} }}} \hfill \\ \vdots \hfill & \ddots \hfill & \vdots \hfill \\ {\frac{{\exp (y_{{l1}} )}}{{\sum\nolimits_{{r = 1}}^{q} {\exp (y_{{lr}} )} }}} \hfill & \cdots \hfill & {\frac{{\exp (y_{{lq}} )}}{{\sum\nolimits_{{r = 1}}^{q} {\exp (y_{{lr}} )} }}} \hfill \\ \end{array} } \right] \in \mathbb{R}^{{l \times q}}$$

### Learning algorithm of dynamic learning network

Neural dynamic learning algorithm (NDLA) has been favored in recent years owing to more rapid convergence and higher precision^40,45^. Its design formula is expressed as

10 $$\begin{aligned} {\dot{e}}(t)=-\lambda {\varPhi }(e(t)). \end{aligned}$$

In Eq. (10), $t\in {\mathbb {R}}$ denotes continuous time. $e(t)\in {\mathbb {R}}$ denotes deviation between prediction output and expectation over *t*. ${\dot{e}}(t)$ denotes the derivative of the deviation *e*(*t*) with respect to *t*. $\lambda >0,\lambda \in {\mathbb {R}}$ denotes NDLA parameter. ${\varPhi }(\cdot )$ denotes mapping function that is a monotonically increasing and odd function^38,40,43,45^. NDLA is conducted in digital computer. Such serial time expression (i.e., Eq. (10)) is transformed into a discrete ones combined with Euler discrete formula^46^, which is shown below.

11 $$\begin{aligned} \frac{e(k+1)-e(k)}{{\tilde{h}}}=-\lambda {\varPhi }(e(k)) \end{aligned}$$

where ${\tilde{h}}>0,{\tilde{h}}\in {\mathbb {R}}$ denotes the discrete step, and $k>0,k\in {\mathbb {Z}}$ denotes discrete time and the $k{\text {th}}$ training round. Equation (11) is further transformed as

12 $$\begin{aligned} e(k+1)=e(k)-\alpha {\varPhi }(e(k))\in {\mathbb {R}} \end{aligned}$$

where $\alpha ={\tilde{h}}\lambda >0,\alpha \in {\mathbb {R}}$ denotes NDLA design coefficient. Thus, Eq. (12) is a discrete NDLA design formula.

#### Theorem 1

*A deviation based learning rule is formulated as a discrete NDLA design formula, i.e.,*$e(k+1)-e(k)=-\alpha {\varPhi }(e(k))$, *where*$\alpha >0$*is a proper value within a range. If the mapping function*${\varPhi }(\cdot )$*in NDLA is a monotonically increasing and odd function, then the deviation between prediction output and expectatione*(*k*) *shows absolute convergence to zero along with discrete timekincreasing.*

#### *Proof*

The discrete NDLA design formula is expressed as

13 $$\begin{aligned} e(k+1)-e(k)=-\alpha {\varPhi }(e(k)). \end{aligned}$$

If the mapping function ${\varPhi }(\cdot )$ is a monotonically increasing and odd function, ${\varPhi }(e(k))>0$ is obtained when the deviation *e*(*k*) satisfies $e(k)>0$. Because NDLA coefficient satisfies $\alpha >0$, $-\alpha {\varPhi }(e(k))<0$ is then deduced, which leads to $e(k+1)<e(k)$ according to Eq. (13). Conversely, when the deviation $e(k)<0$, $-\alpha {\varPhi }(e(k))>0$ is obtained, and $e(k+1)>e(k)$ can be deduced further. Thus, $\left| e(k+1)\right| <\left| e(k)\right|$ is deduced. With the discrete time *k* increasing, the absolute value of the deviation will absolutely converge to zero, which is expressed as $\lim \limits _{k\rightarrow +\infty }|e(k)|=0$. The proof is completed. $\square$

For the mapping function, the linear, tanh and sinh types are applied as examples in this paper, i.e.,

14 $$\begin{aligned} {\varPhi }_{1}(z)= & {} z \end{aligned}$$

15 $$\begin{aligned} {\varPhi }_{2}(z)= & {} \tanh (z) \end{aligned}$$

16 $$\begin{aligned} {\varPhi }_{3}(z)= & {} \sinh (z). \end{aligned}$$

**Figure 2:**
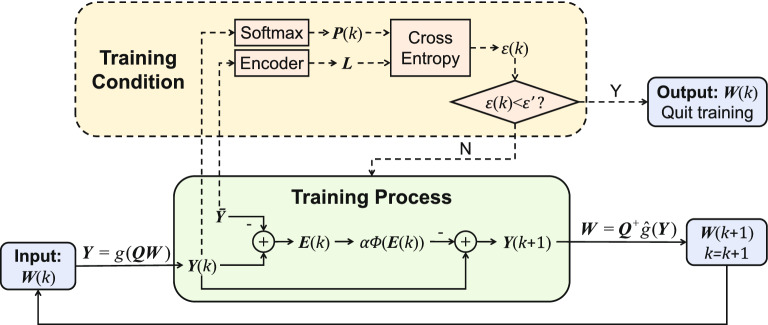
Diagram of neural dynamic learning algorithm (NDLA). If training error $\varepsilon (k)$ calculated by the class possibility matrix ${{\varvec{P}}}(k)$ and the label matrix ***L*** satisfies $\varepsilon (k)\ge \varepsilon '$, where $\varepsilon '$ denotes a threshold, the weight matrix connecting between hidden and output layers ${{\varvec{W}}}(k)$ will be updated as ${{\varvec{W}}}(k+1)$ at the $(k+1){\text {th}}$ training round under the situation of known NDLA design coefficient $\alpha$, mapping function ${\varPhi }(\cdot )$, hidden output matrix ${{\varvec{Q}}}$, the diagnosis deviation matrix ${{\varvec{E}}}$, predicted diagnosis output matrix ${{\varvec{Y}}}$, and the label matrix ${\varvec{\bar{Y}}}$.

The discrete NDLA design formula in this paper is used to train dynamic learning network, in which the weight matrix ${{\varvec{W}}}$ is iterated. As shown in Fig. 2, when the $k{\text {th}}$ training round is finished, the diagnosis deviation matrix ${\varvec{E}}(k)$ is calculated by

17 $$\begin{aligned} {\varvec{E}}(k)={{\varvec{Y}}}(k)-{\varvec{\bar{Y}}}\in {\mathbb {R}}^{l\times q} \end{aligned}$$

where ${\varvec{Y}}(k)$ denotes output through dynamic learning network and ${\varvec{\bar{Y}}}$ denotes labels of samples in $-1$-1 format. Before the next round, the training error $\varepsilon (k)$ is calculated by cross entropy formula at first, i.e.,

18 $$\begin{aligned} \varepsilon (k)=\frac{1}{l}\sum _{s=1}^{l}\sum _{r=1}^{q}(-L_{sr}\log (P_{sr}))\in {\mathbb {R}} \end{aligned}$$

where $L_{sr}\in {\varvec{{L}}}$ and $P_{sr}\in {{\varvec{P}}}(k)$. The class probability matrix ${{\varvec{P}}}(k)$ is calculated according to Eq. (9). Suppose the threshold of training error is $\varepsilon '\in {\mathbb {R}}$. If $\varepsilon (k)<\varepsilon '$, stop and quit this training process. Otherwise, continue updating ${{\varvec{W}}}$.

For training details of dynamic learning network in the $(k+1){\text {th}}$ training round, the diagnosis deviation matrix ${\varvec{E}}(k+1)$ is obtained by the following equation, i.e.,

19 $$\begin{aligned} {\varvec{E}}(k+1)={\varvec{E}}(k)-\alpha {\varPhi }({\varvec{E}}(k))\in {\mathbb {R}}^{l\times q} \end{aligned}$$

where

20 $$\begin{aligned} {\varvec{E}}(k+1)={{\varvec{Y}}}(k+1)-{\varvec{\bar{Y}}}\in {\mathbb {R}}^{l\times q}. \end{aligned}$$

When ${\varPhi }(\cdot )$ is a monotonically increasing and odd function, ${\varvec{E}}(k)$ can rapidly converge to $\mathbf{0}$ along with *k* increasing according to Theorem 1, which indicates that $\lim \limits _{k\rightarrow +\infty }{{\varvec{Y}}}(k)={\varvec{\bar{Y}}}$ according to Eq. (17). Through softmax formula (i.e., Eq. (9)) and encoder, $\lim \limits _{k\rightarrow +\infty }{{\varvec{P}}}(k)={\varvec{\bar{P}}}$ is obtained, where ${\varvec{\bar{P}}}$ denotes a constant targeted class probability matrix. Thus, the training error $\varepsilon (k)$ satisfies $\lim \limits _{k\rightarrow +\infty }\varepsilon (k)={\tilde{\varepsilon }}$ according to Eq. (18), which indicates that $\varepsilon (k)$ theoretically converges to a targeted constant ${{\tilde{\varepsilon }}}$ along with *k* increasing.

Substituting Eqs. (17) and (20) to Eq. (19), we can obtain discrete neural dynamic equation

21 $$\begin{aligned} {{\varvec{Y}}}(k+1)={{\varvec{Y}}}(k)-\alpha {\varPhi }({\varvec{E}}(k))\in {\mathbb {R}}^{l\times q} \end{aligned}$$

where

22 $$\begin{aligned} {{\varvec{Y}}}(k)= & {} g ({{\varvec{Q}}}{{\varvec{W}}}(k))\in {\mathbb {R}}^{l\times q} \end{aligned}$$

23 $$\begin{aligned} {{\varvec{Y}}}(k+1)= & {} g ({{\varvec{Q}}}{{\varvec{W}}}(k+1))\in {\mathbb {R}}^{l\times q}. \end{aligned}$$

Therefore, ${{\varvec{W}}}(k+1)$ can be figured out under the situation of known ${{\varvec{Q}}}$ and ${{\varvec{W}}}(k)$. Substituting Eqs. (22) and (23) to Eq. (21), we can obtain

24 $$\begin{aligned} g ({{\varvec{Q}}}{{\varvec{W}}}(k+1))=g ({{\varvec{Q}}}{{\varvec{W}}}(k))-\alpha {\varPhi }({\varvec{E}}(k))\in {\mathbb {R}}^{l\times q}. \end{aligned}$$

${{\varvec{W}}}(k+1)$ is figured out by solving Eq. (24) as

25 $$\begin{aligned} {{\varvec{W}}}(k+1)={{\varvec{Q}}}^{+}{} {g} ^{-1}(g ({{\varvec{Q}}}{{\varvec{W}}}(k))-\alpha {\varPhi }({\varvec{E}}(k)))\in {\mathbb {R}}^{n\times q}. \end{aligned}$$

Equation (25) shows the iterated relation between ${{\varvec{W}}}(k+1)$ and ${{\varvec{W}}}(k)$. A training round is thus finished. Without loss of generality, the softsign function is applied as an example of ${g} (\cdot )$. However, the inverse function of softsign function can not be found. Under this circumstance, an approximate expression ${\hat{g}}(\cdot )$ is used instead, i.e.,

26 $$\begin{aligned} \textit{g}^{-1}(z)\approx {\hat{g}}(z)=\frac{z}{1-|z|}. \end{aligned}$$

### Bagging dynamic learning network classifier

To further enhance the generalization of the dynamic learning network, bootstrap aggregating (bagging) algorithm is utilized to construct a more robust bagging dynamic learning network classifier, as illustrated in Fig. 1. In bagging algorithm, some of the samples are randomly collected from all the training features as a subset, which is used to train a dynamic learning network. This process is repeated for several times, and several trained dynamic learning networks are obtained. In combination strategy, these trained dynamic learning networks start by predicting testing samples. Then with regard to these predicted results, the final belonging class is determined based on the principle of plurality voting, i.e., the majority rule^43,47^. Specifically, we initialize a zero vector ${{\varvec{a}}}_{t}=\left[ {a_{t1}},{\ldots },{a_{tq}}\right] \in {\mathbb {R}}^{1\times q}$ for the vote statistic. If the ${\hat{k}}{\text {th}}$ dynamic learning network model determines that the testing sample ${{\varvec{x}}}_{t}=\left[ {x_{t1}},{\ldots },{x_{tm}}\right] \in {\mathbb {R}}^{1\times m}$ belongs to the ${\hat{r}}{\text {th}}$ class (${\hat{k}}\in [1,N],{\hat{r}}\in [1,q];{\hat{k}},{\hat{r}}\in {\mathbb {Z}}$), $a_{t{\hat{r}}}$ will be increased by one. Based on the majority rule, we determine that the index corresponding to the maximum number of votes among all the elements in ${{\varvec{a}}}_{t}$ is the final predicted class after vote statistic.

**Figure 3:**
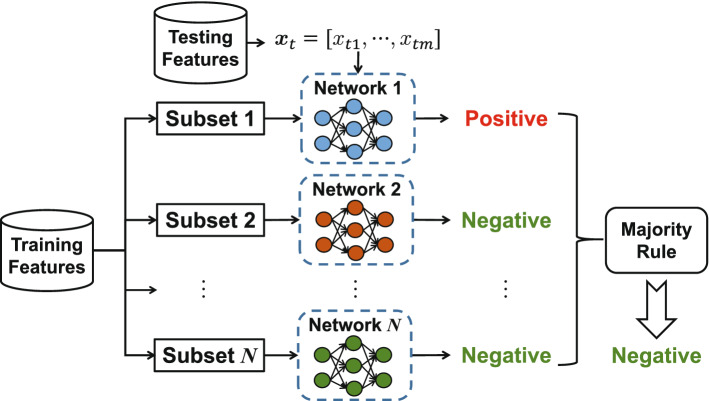
Concrete schematic of bagging dynamic learning network classifier in Fig. 1. *N* dynamic learning networks trained by *N* different types of subsets randomly under-sampled from training features predict the class to which testing images belong, and the final diagnosis result based on these predictions for the testing sample ${{\varvec{x}}}_{t}=\left[ {x_{t1}},{\ldots },{x_{tm}}\right] \in {\mathbb {R}}^{1\times m}$ is determined by the majority rule.

Structure and principle of bagging dynamic learning network classifier are shown in Fig. 3, where dynamic learning networks trained by NDLA using various mapping functions ${\varPhi }(\cdot )$ are simultaneously trained by using various subsets drawn randomly from shuffled training features. Suppose there are two kinds of diagnosis results (positive and negative) and *N* dynamic learning networks. When ${{\varvec{x}}}_{t}$ is entered into this proposed bagging dynamic learning network, according to the majority rule, these dynamic learning networks mutually independently judge the kind of diagnosis result to which it belongs, and then determine the final diagnosis result. For example, when the predicted result of the first dynamic learning network is positive (COVID-19) while those of others are negative (normal), the final diagnosis result is determined as negative. If the vote is tied for a practical COVID-19 screening system, the final result is directly diagnosed as positive, which can effectively reduce the risk of missed diagnosis by enhancing the sensitivity^43^. When the final diagnosis result shows positive, more detailed detections and corresponding treatments must be implemented.

## Results

**Figure 4:**
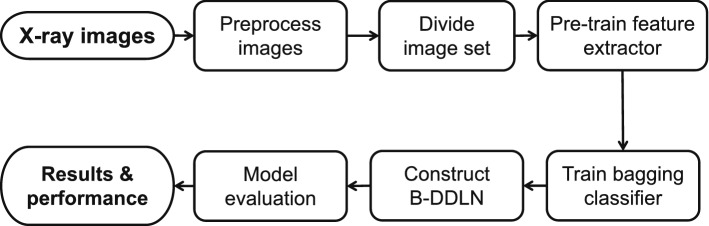
Flowchart of the entire experiment. Preprocessed X-ray chest radiography images are divided into training and testing sets. The training images are used to pre-train convolution blocks as a feature extractor, and a bagging dynamic learning network classifier is subsequently trained to construct B-DDLN diagnosis model. Testing images are used to evaluate the proposed B-DDLN to obtain diagnosis results and corresponding performance.

In this section, the application of the proposed B-DDLN to diagnose the presence of COVID-19 by analyzing X-ray chest radiography images is described. This description consists of six parts: description and preprocessing of image set, feature extraction, analysis and random under-sampling of samples, experimental results of the proposed B-DDLN, and comparisons between B-DDLN and other diagnosis models. Figure 4 shows a flowchart of the entire experiment where the feature extractor and bagging classifier are successively trained using training images and corresponding features, respectively, to construct the B-DDLN diagnosis model. Testing images are used to evaluate this proposed B-DDLN diagnosis model and the diagnosis results are obtained.

### Description and preprocessing of the image set

**Figure 5:**
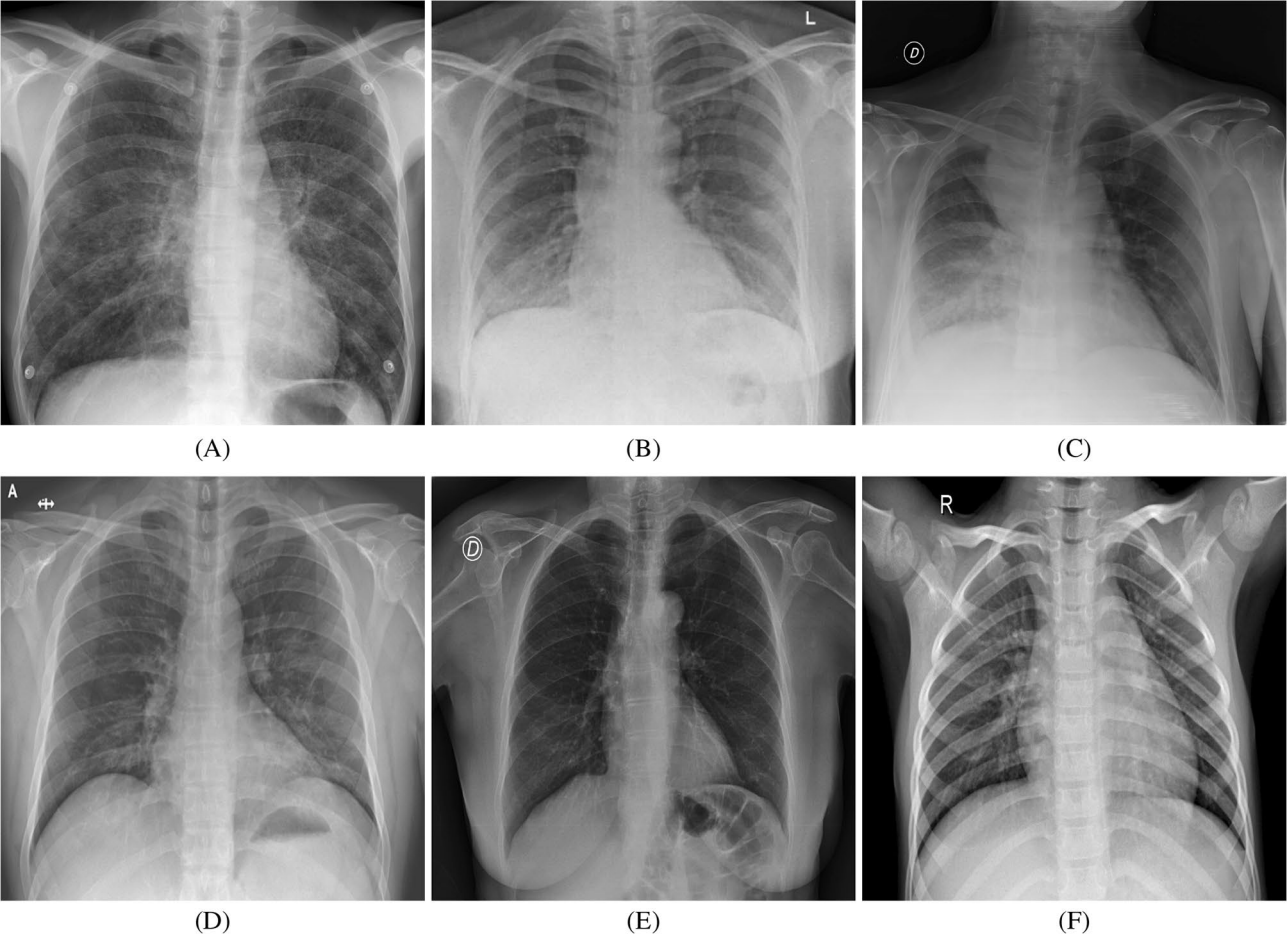
Examples of X-ray chest radiography images collected from COVID-19 patient and normal cases. (**A**) A COVID-19 patient case from COVID-19 open image data collection. (**B**) A COVID-19 patient case from Fig. 1 COVID-19 Chest X-ray Dataset Initiative. (**C**) A COVID-19 patient case from ActualMed COVID-19 chest X-ray dataset initiative. (**D**) A COVID-19 patient case from COVID-19 Radiography Database. (**E**) A normal case from ActualMed COVID-19 Chest X-ray dataset initiative. (**F**) A normal case from COVID-19 radiography database.

In this study, we combine and modify the four data repositories to create the COVID-19 image set with binary-classification by leveraging the following types of patient and normal cases from each of the data repositories:

- COVID-19 patient cases from COVID-19 open image data collection, which was created by assembling medical images from websites and publications^48^. This data collection currently contains hundreds of frontal-view X-ray images, which is a necessary resource to develop and evaluate tools to aid in the diagnosis of COVID-19^49^. An example is shown in Fig. 5A.
- COVID-19 patient cases from Fig. 1 COVID-19 Chest X-ray Dataset Initiative, which are built to enhance diagnosis models for COVID-19 disease detection and risk stratification. An example is shown in Fig. 5B.
- COVID-19 patient and normal cases from ActualMed COVID-19 Chest X-ray Dataset Initiative. A COVID-19 example is shown in Fig. 5C and a normal example is shown in Fig. 5E.
- COVID-19 patient and normal cases from COVID-19 Radiography Database (winner of the COVID-19 data set award by Kaggle community), which is created by a team of researchers from Qatar University, Doha, Qatar and the University of Dhaka, Bangladesh along with their collaborators from Pakistan and Malaysia in collaboration with medical doctors. A COVID-19 example is shown in Fig. 5D and a normal example is shown in Fig. 5F.

After gathering X-ray chest radiography images from these collections, there are 2284 images in the newly formed image set (816 from COVID-19 patient cases and 1468 from normal cases). Preprocessing steps of X-ray chest radiography images are necessary for improving the quality of image data and enhancing image features. First of all, image channel sequence is changed from BGR to RGB. Secondly, the sizes of all the images are uniformly set as $224\times 224\times 3$. Thirdly, these images are labeled for training B-DDLN. After the division of image set, 180 testing images (100 from normal and 80 from COVID-19 patient cases) among all are selected to evaluate the proposed B-DDLN.

### Feature extractor pre-training

The feature extractor of images is pre-trained in an environment equipped with PyCharm Community Edition of 2020.1.2 version and MATLAB 2020a on a personal computer with an Intel Core i7 processor, NVIDIA GeForce GTX 1660 Ti GPU architecture and 32 GB RAM running under Windows 10 operating system. We adopt scikit-learn, TensorFlow 2.2.1 and keras to build convolution blocks and pre-train them. When these convolution blocks finish pre-training as a feature extractor $h(\cdot )$, a $224\times 224\times 3$ preprocessed X-ray chest radiography image tensor ${\varvec{g}}_{s}$ is input into $h(\cdot )$, and its corresponding 512-dimension extracted feature vector $h({\varvec{g}}_{s})$ can be obtained as output. Therefore, the features extracted from training images are utilized to train the bagging dynamic learning network classifier, and the features extracted from testing images are used to evaluate this bagging dynamic learning network classifier.

### One-way analysis of variance

**Figure 6:**
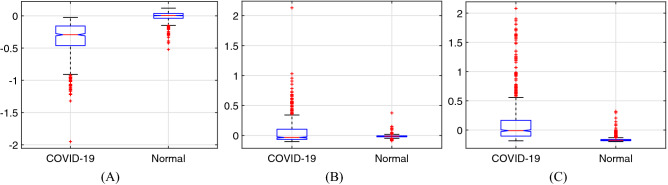
Box-plots among training feature samples in the previous three dimensions by one-way ANOVA. (**A**) The $1{\text {st}}$ dimension. (**B**) The $2{\text {nd}}$ dimension. (**C**) The $3{\text {rd}}$ dimension.

To investigate whether different values of an attribute have a significant effect on the category, one-way analysis of variance (one-way ANOVA) is carried out by using the features extracted from training images in this study. Each training feature sample is a 512-dimension vector. The analytic results of the previous three dimensions by one-way ANOVA are obtained as examples, which are listed in Table 1. Corresponding box-plots are shown in Fig. 6. It can be seen from Table 1 that the *p*-value is much smaller than 0.05, which suggests that a correlation exists between these three dimensions of features and sample category. The results of *F*-value and *p*-value in Table 1 and Fig. 6 collectively substantiate the existence of significant differences among training feature samples belonging to different categories.

**Table 1:** Analytic results among training feature samples in the previous three dimensions by one-way ANOVA.

Dimension	Variation source	Square sum (SS)	Degree of freedom (df)	SS/df	*F*-value	*p*-value
1st	Groups	55.563	1	55.6625	2155.96	0
	Error	54.269	2102	0.0258	–	–
	Total	109.932	2103	–	–	–
2nd	Groups	2.2625	1	2.2625	160.75	1.49852 × 10^−35^
	Error	29.5846	2102	0.01407	–	–
	Total	31.8471	2103	–	–	–
3rd	Groups	39.553	1	39.5534	773.36	3.35758 × 10^−145^
	Error	107.506	2102	0.0511	–	–
	Total	147.06	2103	–	–	–

### Random under-sampling of image features

After division of the image set, there are 2104 left in the training images, among which 736 come from COVID-19 patient and 1368 from normal cases. Such distribution causes category imbalance, which hinders the classifier from releasing effective performance. To overcome this problem, random under-sampling of samples (i.e., feature vectors) is necessary, which means that 1000 samples from COVID-19 patient cases are randomly selected among all. In this study, we perform this random under-sampling three times and obtain three different subsets. Each subset consists of 1736 samples, with 736 come from COVID-19 patient cases and 1000 from normal cases. By using these three subsets, three dynamic learning networks (i.e., $N=3$) with three different types of mapping functions are trained. As examples, these three types of mapping functions are: linear (i.e., Eq. (14)), tanh (i.e., Eq. (15)) and sinh (i.e., Eq. (16)) types. Furthermore, the bagging dynamic learning network classifier is constructed by these three dynamic learning networks and bagging algorithm.

### Classification performance of B-DDLN

**Figure 7:**
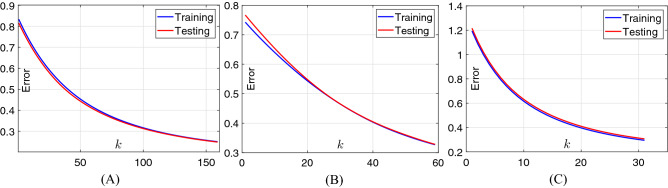
Trends of errors for training and testing dynamic learning network (DLN) in case 1 as the training round *k* increases. (**A**) DLN trained by NDLA with linear type of mapping function. (**B**) DLN trained by NDLA with tanh type of mapping function. (**C**) DLN trained by NDLA with sinh type of mapping function.

**Figure 8:**
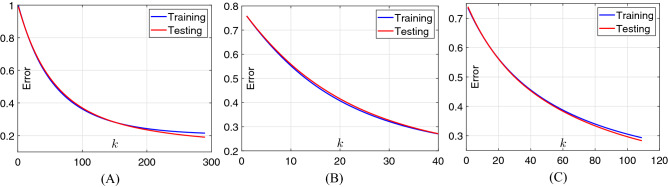
Trends of errors for training and testing dynamic learning network (DLN) in case 2 as the training round *k* increases. (**A**) DLN trained by NDLA with linear type of mapping function. (**B**) DLN trained by NDLA with tanh type of mapping function. (**C**) DLN trained by NDLA with sinh type of mapping function.

In this study, two cases of activation functions are designed in the dynamic learning network. In case 1, all the activation functions in the hidden and output neurons are set as softsign function (i.e., Eq. (6)). In case 2, activation functions in the output neurons are also designed as softsign function while those in the hidden neurons are set as power-softsign function (i.e., Eq. (7)). For classifying the extracted features from convolution blocks of B-DDLN, trends of classification errors calculated according to cross entropy formula (i.e., Eq. (18)) of dynamic learning networks trained by NDLA in two cases are shown in Figs. 7 and 8 . From Figs. 7 and 8 , we can see that both two tendencies monotonically and rapidly decrease and even converge to a specific minor value when the training round *k* is large enough. The testing errors $\varepsilon _{t}$ in all of the subgraphs are always decreasing as *k* increases. These phenomena indicate that the dynamic learning networks trained by NDLA with different mapping and activation functions are not over-fitting. They also verify that using NDLA can rapidly make the classification error monotonously converge and enhance precision. In the testing process, confusion matrices of the proposed B-DDLNs under both two cases of activation functions are listed in Table 2, where

- True (T): Actual result is normal.
- False (F): Actual result is COVID-19.
- Positive (P): Predicted result is normal.
- Negative (N): Predicted result is COVID-19.

**Table 2:** Confusion matrices of the proposed B-DDLN.

Activation function	Model	Confusion matrix		Accuracy (%)
Case 1	B-DDLN	TP = 99	FN = 1	98.8889
		FP = 1	TN = 79	
Case 2	B-DDLN	TP = 99	FN = 1	98.8889
		FP = 1	TN = 79	

Thus, the testing accuracy is calculated by Eq. (27), i.e.,

27 $$\begin{aligned} \text {Accuracy}=\frac{\text {TP+TN}}{\text {TP+TN+FP+FN}}. \end{aligned}$$

**Figure 9:**
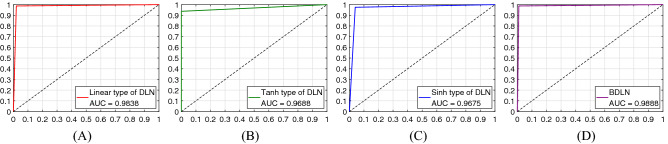
Receiver operating characteristic curves (ROC curves) of dynamic learning networks (DLN) and bagging dynamic learning network (BDLN) in case 1. (**A**) DLN trained by NDLA with linear type of mapping function. (**B**) DLN trained by NDLA with tanh type of mapping function. (**C**) DLN trained by NDLA with sinh type of mapping function. (**D**) Bagging dynamic learning network (BDLN) classifier.

**Figure 10:**
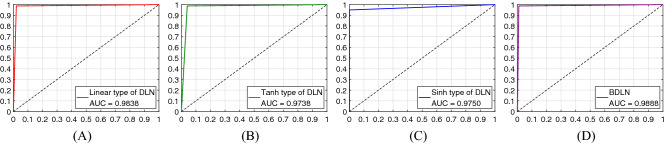
Receiver operating characteristic curves (ROC curves) of dynamic learning networks (DLN) and bagging dynamic learning network (BDLN) in case 2. (**A**) DLN trained by NDLA with linear type of mapping function. (**B**) DLN trained by NDLA with tanh type of mapping function. (**C**) DLN trained by NDLA with sinh type of mapping function. (**D**) Bagging dynamic learning network (BDLN) classifier.

In Table 2, most testing samples distribute in the TP and TN areas, which illustrates that the proposed B-DDLNs in two cases achieve high diagnosis accuracy. The accuracies of dynamic learning networks with corresponding hyper-parameters in two cases are listed in Table 3. In the process of training the proposed dynamic learning network, the proper sets of hyper-parameters are adjusted and selected by 10-fold cross validation, which makes the testing performance including accuracy reach the best level. Early stopping is also adopted, which can avoid over-fitting. From Table 3, we can see that the highest testing accuracy of a single dynamic learning network can reach $98.3333\%$ in a short time. After plurality voting, the bagging dynamic learning networks in two cases achieve $98.8889\%$ accuracy, which shows good precision for diagnosing COVID-19. The number of hidden neurons is no more than 60, which suggests that the dynamic learning network is a lightweight network. For the testing set, the receiver operating characteristic curves (ROC curves) of the dynamic learning network and its improved models, i.e., bagging dynamic learning network classifiers, under two cases are shown in Figs. 9 and 10 . It can be seen that these ROC curves approach coordinate point (0, 1), which leads to large corresponding areas under curves (AUC). These phenomena further substantiate the superior classification performance of the proposed B-DDLN diagnosis model.

**Table 3:** Accuracies and hyper-parameters of single dynamic learning networks.

Activation function	Accuracy and hyper-parameters	Mapping function type		
		Linear	Tanh	Sinh
Case 1	Testing accuracy (%)	98.3333	97.2222	96.6667
	Training time (*s*)	6.0156	2.3906	1.4688
	Threshold ${\varepsilon}^{\prime}$	0.2506	0.3270	0.2947
	Training rounds *k*	159	59	31
	NDLA coefficient $\alpha$	0.01	0.02	0.04
	Hidden neurons *n*	50	55	60
Case 2	Testing Accuracy (%)	98.3333	97.2222	97.7778
	Training time (*s*)	17.5000	2.7344	6.2344
	Threshold ${\varepsilon}^{\prime}$	0.2154	0.2711	0.2935
	Training rounds *k*	290	40	109
	NDLA coefficient $\alpha$	0.01	0.04	0.01
	Hidden neurons *n*	42	40	41

### Comparison analysis

In order to further verify the superiority of the proposed B-DDLN diagnosis model, fair and comprehensive comparisons in classification performance between B-DDLN and other existing methods on the same image set are presented in this section.

For comparisons on the same application domain, we use the same images and 10-fold cross validation to train some state-of-the-art methods under the same operating environment, and we apply the same testing images to evaluate them. Testing accuracy is used as the main indicator to measure the classification performance of deep learning models, whose results are listed in Table 4. In Table 4, testing accuracy of the proposed B-DDLN is an average value, while those of others show the highest values. From Table 4, we can see that the testing accuracy of the proposed B-DDLN reaches the top level when compared with other deep learning models, which manifests the superior precision of the proposed B-DDLN in diagnosing COVID-19. In addition, the structure of proposed B-DDLN is simpler and more lightweight than most of the existing state-of-the-art models, which suggests that B-DDLN is more easily deployed in a medical software system with a higher speed of diagnostic prediction.

**Table 4:** Comparison results between B-DDLN and some existing state-of-the-art methods.

Model/method	Testing accuracy ($\%$)
**The proposed B-DDLN (case 1)**	**98.8889**
**The proposed B-DDLN (case 2)**	**98.8889**
ResNet	97.2222
CNN-X (transfer learning)^16^	95.0000
ResNet-based multi-channel transfer learning model^50^	93.8889
DarkCovidNet^51^	97.7778
SPEA-II-based modified AlexNet^52^	98.3333
Ensemble densely connected convolutional neural network^53^	97.2222
Ensemble deep transfer learning model^54^	96.1111

The superior performance of the proposed B-DDLN diagnosis model are highlighted in bold.

For the same extracted image features, comparison results in classification performance between the proposed B-DDLN, especially its bagging dynamic learning network, and other classifiers are listed in Table 5, where the corresponding experiments are conducted under the same conditions. From Table 5, we can see that the specificity and sensitivity of the proposed B-DDLN are 0.9875 and 0.9900, respectively, which indicates that misdiagnosis and missed diagnosis of COVID-19 are very rare. Most of the evaluation indicators simultaneously remain the highest among all the classifiers. These findings substantiate the excellent advantages and superiorities of the proposed B-DDLN in diagnosing the presence of COVID-19.

**Table 5:** Comparison results between B-DDLN and other classifiers for classifying the same extracted image features.

Classifier	Testing accuracy(%)	F1-score	Precision	Specificity	Sensitivity	Kappa value	AUC
**B-DDLN1**$^{\text{ a }}$	**98.8889**	**0.9900**	**0.9900**	**0.9875**	**0.9900**	**0.9775**	**0.9888**
**B-DDLN2**$^{\text{ b }}$	**98.8889**	**0.9900**	**0.9900**	**0.9875**	**0.9900**	**0.9775**	**0.9888**
$\hbox {BPNN}^{\text{ c }}$	95.5556	0.9588	0.9894	0.9875	0.9300	0.9107	0.9587
Naive Bayesian	92.7778	0.9353	0.9307	0.9125	0.9400	0.8536	0.9012
$\hbox {LDA}^{\text{ d }}$	93.3333	0.9406	0.9314	0.9125	0.9500	0.8647	0.9313
$\hbox {QDA}^{\text{ e }}$	94.4444	0.9485	0.9787	0.9750	0.9200	0.8883	0.9475
Linear $\hbox {SVM}^{\text{ f }}$	96.6667	0.9697	0.9796	0.9750	0.9600	0.9327	0.9675
Gaussian SVM	95.5556	0.9600	0.9600	0.9500	0.9600	0.9100	0.9550
*k*-$\hbox {NN}^{\text{ g }}$	95.0000	0.9569	0.9174	0.8875	1.0000	0.8976	0.9437
Logistic regression	90.5556	0.9171	0.8952	0.8625	0.9400	0.8075	0.9012
Decision tree	93.3333	0.9394	0.9490	0.9375	0.9300	0.8653	0.9337
Bagging tree	92.7778	0.9366	0.9143	0.8875	0.9600	0.8528	0.9237
Boosting tree	96.1111	0.9648	0.9697	0.9625	0.9600	0.9213	0.9612

The superior performance of the proposed B-DDLN diagnosis model are highlighted in bold.

^a^B-DDLN in case 1.

^b^B-DDLN in case 2.

^c^Back propagation neural network.

^d^Linear discriminant analysis.

^e^Quadratic discriminant analysis.

^f^Support vector machine.

^g^$$k$$-nearest neighbor.

## Discussion

From the experimental and compared results, we can see that using the proposed B-DDLN, which consists of a feature extractor and a bagging dynamic learning network classifier, achieves the best diagnostic performance compared with other state-of-the-art models on the same application domain. The proposed B-DDLN is trained and constructed in a short time, and shows superior classification performance without over-fitting. The diagnosis accuracy for distinguishing COVID-19 or normal stays at the top level when using the proposed B-DDLN owing to the existence of NDLA and bagging algorithm. The proposed B-DDLN diagnosis model is so lightweight that it can be deployed as a medical software system under the restrictions of high real-time requirement and limited hardware resources.

However, the proposed B-DDLN has certain limitations, and some improvements can be made. First of all, the extracted features of X-ray chest radiography images are not detailed and separable enough. The feature extractor needs to be improved by remolding and optimizing the structure and pre-training method. Secondly, the forms of labels for input images, i.e., ${\varvec{\bar{Y}}}$ and ${{\varvec{L}}}$, are not unified, which impedes training dynamic learning networks. Thirdly, the calculation method of class possibility needs to be improved to enhance confidence. Furthermore, the proposed B-DDLN should be used for diagnosing different types of diseases to verify its generalization performance.

## Conclusion

In this paper, B-DDLN has been proposed for diagnosing presence of COVID-19 using X-ray chest radiography images. After some preprocessing steps, training images have been used to pre-train convolution blocks as a feature extractor. For the training features extracted from this extractor, a bagging dynamic learning network classifier based on neural dynamic learning algorithm (NDLA) and bagging algorithm has been trained. Experimental results have verified that the proposed B-DDLN diagnosis model possesses high training efficiency, and has considerably increased diagnosis accuracy under the restrictions of high real-time requirement and limited hardware resources. The diagnosis accuracy of the proposed B-DDLN has reached $98.8889\%$, which is the highest among all the existing state-of-the-art methods. In addition, corresponding performance indicators of bagging dynamic learning network classifier have reached the top level among all, further substantiating that the proposed B-DDLN possesses excellent diagnostic ability. Such accurate diagnosis outcomes provide assertive evidences in early detection of COVID-19, which helps to provide suitable treatments for patients and maintain human health.

In the future, the proposed B-DDLN diagnosis model can be trained for diagnosing not only the presence of COVID-19, but also other types of pneumonia such as SARS and community-acquired pneumonia, which cannot be detected by nucleic acid amplification testing alone. For excellent diagnostic performance, a medical software system based on the proposed B-DDLN diagnosis model can be developed for diagnosing multi-classification of pneumonia by automatically recognizing X-ray chest radiography images, generating diagnostic reports, and providing corresponding therapeutic schemes for diagnostic results. Such a fully functional system would be extremely useful for clinicians.

## Data Availability

COVID-19 image data collection is available at https://github.com/ieee8023/covid-chestxray-dataset. Figure 1 COVID-19 chest X-ray dataset initiative is available at https://github.com/agchung/Figure1-COVID-chestxray-dataset. Actualmed COVID-19 chest X-ray dataset initiative is available at https://github.com/agchung/Actualmed-COVID-chestxray-dataset. COVID-19 radiography database is available at https://www.kaggle.com/tawsifurrahman/covid19-radiography-database.

## Code availability

Source codes and data set of the current study are available at https://github.com/OliverChan96/B-DDLN.
